# Intestinal obstruction due to phytobezoar induced in the Meckel’s diverticulum-report of two cases

**DOI:** 10.14744/nci.2020.89656

**Published:** 2022-04-01

**Authors:** Necattin Firat, Baris Mantoglu, Fatih Altintoprak, Ali Muhtaroglu, Mertcan Akcay

**Affiliations:** 1 Department of General Surgery, Sakarya Training and Research Hospital, Sakarya, Turkey; 2 Department of General Surgery, Sakarya University Faculty of Medicine, Sakarya, Turkey

**Keywords:** Intestinal obstruction, mechanical ileus, Meckel’s diverticulum, phytobezoar

## Abstract

Meckel’s diverticulum is generally asymptomatic, but it may become symptomatic due to various reasons and maybe the etiology of the acute abdominal syndrome. Bezoars are formed by the combination of non-digestible substances in the gastrointestinal tract, and which are among the rare causes of intestinal obstruction. The formation of bezoars in Meckel’s diverticulum and subsequent intestinal obstruction is a rare condition. In this article, two cases with intestinal obstruction due to bezoar in Meckel’s diverticulum and their surgical treatment had presented.

**B**ezoars are made up of substances taken by mouth, and which cannot be digested and accumulate in the stomach and small intestine. They have been named by different names according to the type of material they contain (e.g., trichobezoar, phytobezoar, and lactobezoar) [1]. Phytobezoars, which are formed by consuming high amounts of fiber foods that are difficult to digest, are the most common type of bezoars and play a role in the etiology of 0.4–4% of intestinal obstructions [2]. Meckel’s diverticulum is the most common (2%) congenital anomaly of the gastrointestinal tract [3]. While it is frequently presented as intestinal obstruction and gastrointestinal bleeding in childhood, it is usually observed asymptomatic in adults and is found incidentally during radiological examinations or abdominal surgeries performed for other reasons [3]. Phytobezoar is a rare condition causing small bowel obstruction in Meckel’s diverticulum, and the literature is composed of a limited number of case reports [4].

## Case Reports

**Figure 1.**
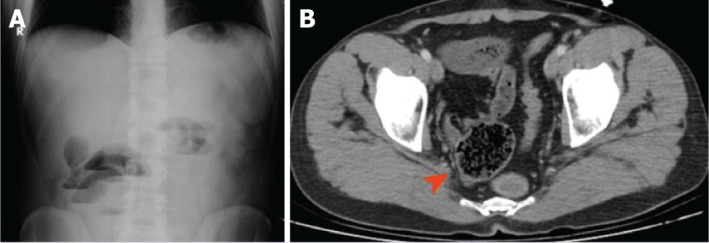
Plain abdominal radiography **(A)**; air-fluid levels consistent with mechanical intestinal obstruction, abdominal computed tomography examination **(B)**; the appearance of a foreign body (bezoar) in the diverticulum of the bowel wall in the small bowel segments matching the ileum, the small bowel dilatation proximal to this level, and the small bowel feces within the dilated small bowel segments just proximal to the obstruction level.

**Figure 2.**
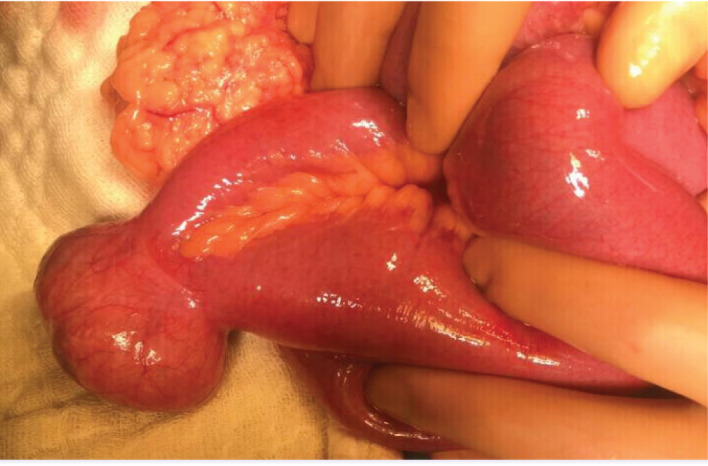
Surgical findings; Meckel’s diverticulum filled with bezoars.

**Case 1 –** A 34-year-old female patient was admitted to the emergency department with complaints of severe abdominal pain and vomiting lasting for 1 day. There was no history of a co-morbid disease or previous abdominal surgery. Hemodynamic parameters were stable at presentation (Blood pressure [BP] 110/70 mm Hg, pulse rate [PR] 68). Physical examination revealed abdominal distention and generalized tenderness, but no rebound was detected. Laboratory investigations were normal, except leukocytosis (11,700/mm^3^). Conventional abdominal X-ray showed air-fluid levels consistent with mechanical intestinal obstruction (Fig. 1A). Abdominal computed tomography revealed a foreign body in the diverticulum of the small intestine at the level of the ileum. Proximal to this level, it was determined that there was thin barred dilatation and that the obstruction level had the appearance of small intestine feces within the dilated small bowel segments just proximal (Fig. 1B). In the abdominal exploration of the patient who underwent emergency surgery with a preliminary diagnosis of mechanical intestinal obstruction; Meckel’s diverticulum was found to be 60 cm proximal the ileocecal valve, and the bezoar in the diverticulum was pressured to the intestinal lumen to cause obstruction (Fig. 2). The patient underwent to diverticulectomy, and during the postoperative follow-up, wound infection developed. The patient discharged on the 10^th^ post-operative day without any problem. Histopathological examination revealed phytobezoar, non-specific chronic inflammation, and Meckel’s diverticulum. The histopathology of faecolith exposed vegetable material.

**Figure 3.**
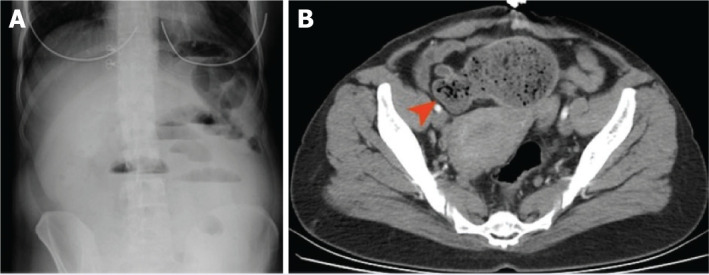
Plain abdominal radiography **(A)**; air-fluid levels consistent with mechanical intestinal obstruction, abdominal computed tomography examination **(B)**; oval-shaped lesion in the small intestine lumen at the level of the ileum, containing air beads (compatible with bezoar), causing obstruction.

**Case 2 –** A 35-year-old male patient was admitted to the emergency department with complaints of severe abdominal pain and vomiting lasting for 2 days. There was no history of a co-morbid disease or previous abdominal surgery. Hemodynamic parameters were stable at presentation (BP 120/80 mm Hg, PR 74). Physical examination revealed abdominal distention and generalized tenderness, but no rebound was detected. Laboratory investigations were normal, except leukocytosis (10.500/mm^3^). Conventional abdominal X-ray showed air-fluid levels consistent with mechanical intestinal obstruction (Fig. 3A). In abdominal CT examination; in the small intestine lumen at the ileum level, an oval-shaped lesion with air beads (compatible with the bezoar) causing obstruction was detected (Fig. 3B). In the abdominal exploration of the patient who underwent emergency surgery with a preliminary diagnosis of mechanical intestinal obstruction; Meckel’s diverticulum was found 60 cm proximal to the ileocecal valve and the bezoar in the diverticulum caused congestion by compressing the intestinal lumen due to the mass effect (Fig. 3). The patient had diverticulectomy, and post-operative follow-up was uneventful. He was discharged on the 5th postoperative day without any complaint. Histopathological examination exposed vegetative bodies and necrotic materials consonant with the diagnosis of phytobezoar. In addition, it was reported that mucosal sloughing, submucosal congested blood vessels, and acute inflammatory exudate in the Meckel’s diverticulum.

## Discussion

Meckel’s diverticulum is the most common congenital anomaly of the gastrointestinal tract and encountered in approximately 2% of the population [3]. Although Meckel’s diverticulum is generally asymptomatic, it may become symptomatic due to intussusception, inflammatory adhesion, or volvulus associated with the omphalomesenteric band and may cause surgical intervention [4]. Intestinal obstruction due to bezoar formation in Meckel’s diverticulum is very rare, and the literature is limited to a few case reports [5]. In both cases, there were Meckel diverticulum between 60 and 80 cm of the ileocecal valve and intestinal obstruction due to bezoar in the diverticulum. It is well known that the most important risk factor for the development of intestinal phytobezoar is excessive consumption of fibrous foods that are difficult to digest.

It has been reported in the literature that a history of gastric surgery such as pyloroplasty or gastrojejunostomy, where the gastric outflow pathway is enlarged, the presence of medical conditions such as hypothyroidism with slowing gastrointestinal tract passage and diabetes mellitus, are factors that facilitate the intestinal phytobezoar formation [6]. On the other side, chewing problems due to deterioration of the tooth structure with advancing age may be considered as a separate risk factor in elderly patients without the aforementioned bezoar formation facilitating factors. Both of the cases, we presented were young, and there were no risk factors for bezoar formation. Since the consumption of fibrous foods which is the most important risk factor for the formation of Bezoar, is quite common in our region (especially Persimmon consumption), that we often encounter intestinal bezoar patients without other facilitating risk factors.

Abdominal CT is quite effective in the diagnosis of intestinal bezoar cases, and sensitivity and specificity rates were reported as 90% and 57% in the presence of obstruction [7]. It is an advantage to be able to detect the presence of other intra-abdominal possible pathologies on CT, but attention should be paid to the possible synchronous bezoar images within the dilated intestinal segments proximal to the level of obstruction in bezoar cases. Therefore, in the literature, cases that required additional surgical intervention for bezoars which have not observed in the obstruction area due to the presence of the bezoar in another part of the dilated intestinal lumen has been reported [8].

### Conclusion

As a result; although bezoars are among the rare causes of intestinal obstruction, they should have considered as the first diagnostic option in the geographic regions in which high fibered food consumption is a tradition. Since the presence of Meckel’s diverticulum constitutes a potential site for bezoar accumulation in the intestinal tract, it should be kept in mind that these two rare conditions may coexist.

## References

[1] Andrus CH, Ponsky JL (1988). Bezoars: classification, pathophysiology, and treatment.. Am J Gastroenterol.

[2] Ripollés T, García-Aguayo J, Martínez MJ, Gil P (2001). Gastrointestinal bezoars: sonographic and CT characteristics.. AJR Am J Roentgenol.

[3] Bani-Hani KE, Shatnawi NJ (2004). Meckel’s diverticulum: comparison of incidental and symptomatic cases.. World J Surg.

[4] Fagenholz PJ, de Moya MA (2011). Laparoscopic treatment of bowel obstruction due to a bezoar in a Meckel’s diverticulum.. JSLS.

[5] Yau KK, Siu WT, Law BK, Cheung HY, Ha JP, Li MK (2005). Laparoscopic approach compared with conventional open approach for bezoar-induced small-bowel obstruction.. Arch Surg.

[6] Erzurumlu K, Malazgirt Z, Bektas A, Dervisoglu A, Polat C, Senyurek G (2005). Gastrointestinal bezoars: a retrospective analysis of 34 cases.. World J Gastroenterol.

[7] Taourel PG, Fabre JM, Pradel JA, Seneterre EJ, Megibow AJ, Bruel JM (1995). Value of CT in the diagnosis and management of patients with suspected acute small-bowel obstruction.. AJR Am J Roentgenol.

[8] Hoover K, Piotrowski J, St Pierre K, Katz A, Goldstein AM (2006). Simultaneous gastric and small intestinal trichobezoars--a hairy problem.. J Pediatr Surg.

